# Factors related to pain after cardiac surgery

**DOI:** 10.1186/cc14661

**Published:** 2015-09-28

**Authors:** Ana Elza O Mendonça, Gilson V Torres, Luciana A Reis, Marina G Salvetti, Thaiza TX Nobre

**Affiliations:** 1 Universidade Federal do Rio Grande do Norte, Lagoa Nova, Natal, Rio Grande do Norte, Brazil

## Introduction

Pain related to heart surgery is a limiting factor for patient recovery and it is moderate to severe in 40-60% of patients.

## Objective

To analyze the risk factors for the presence of pain in patients after cardiac surgery.

## Methods

A prospective study in two hospitals in the city of Natal, RN. The sample consisted of 160 patients undergoing cardiac surgery, 57.5% male, mean age 56.8 years (SD = 14.4). Pain was evaluated by the numerical pain rating scale between days 1 and 5 after surgery. The variables that present p < 0.20 in the bivariate analysis were selected for multivariate analysis by logistic regression to establish a pain model prediction.

## Results

The factors associated with pain in the bivariate analysis were gender, age, race, work, diabetes, obesity, back pain, cardiopulmonary bypass, mediastinal drain, side and mediastinal drain, anesthesia, surgical time, coughing, vomiting, drain time and medication. Multivariate analysis allowed the identification of six risk factors for the occurrence of pain (p < 0.05): surgery lasting more than three hours, side and mediastinal drain, cough, vomiting, drain time for more than 24 hours and being female.

## Conclusion

This study identified risk factors for pain related to heart surgery. Knowing these factors allows enhance care planning in order to prevent and minimize the occurrence of pain in the postoperative period.

